# Supplementary material for: Transcriptome and Metabolome Dynamics Explain Aroma Differences between Green and Red Prickly Ash Fruit
Source: Foods. 2021 Feb 10;10(2):391. doi: 10.3390/foods10020391 (PMC7916813; doi:10.3390/foods10020391)
Supplement: Supplementary file 1 [file foods-10-00391-s001.pdf]

Supplementary Files

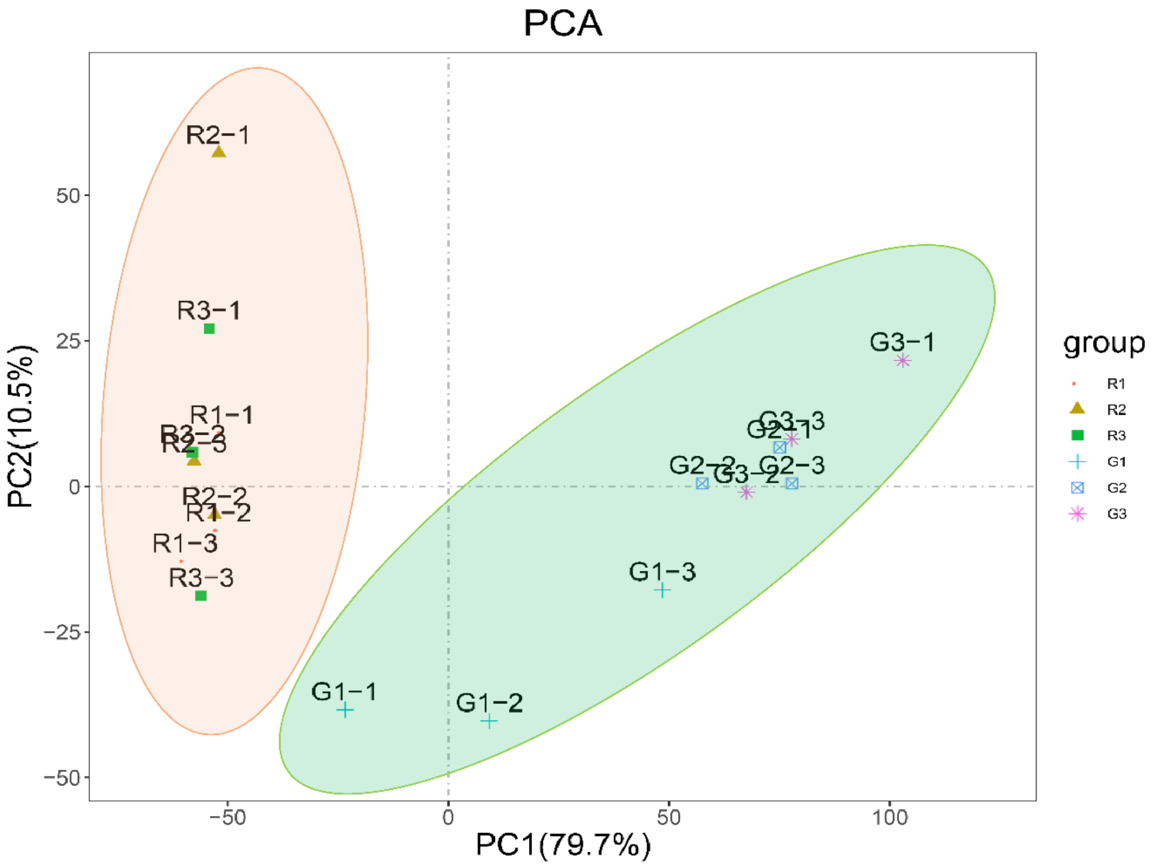

Figure S1. Principal component analysis (PCA) of terpenoid content in green and red prickly ash.

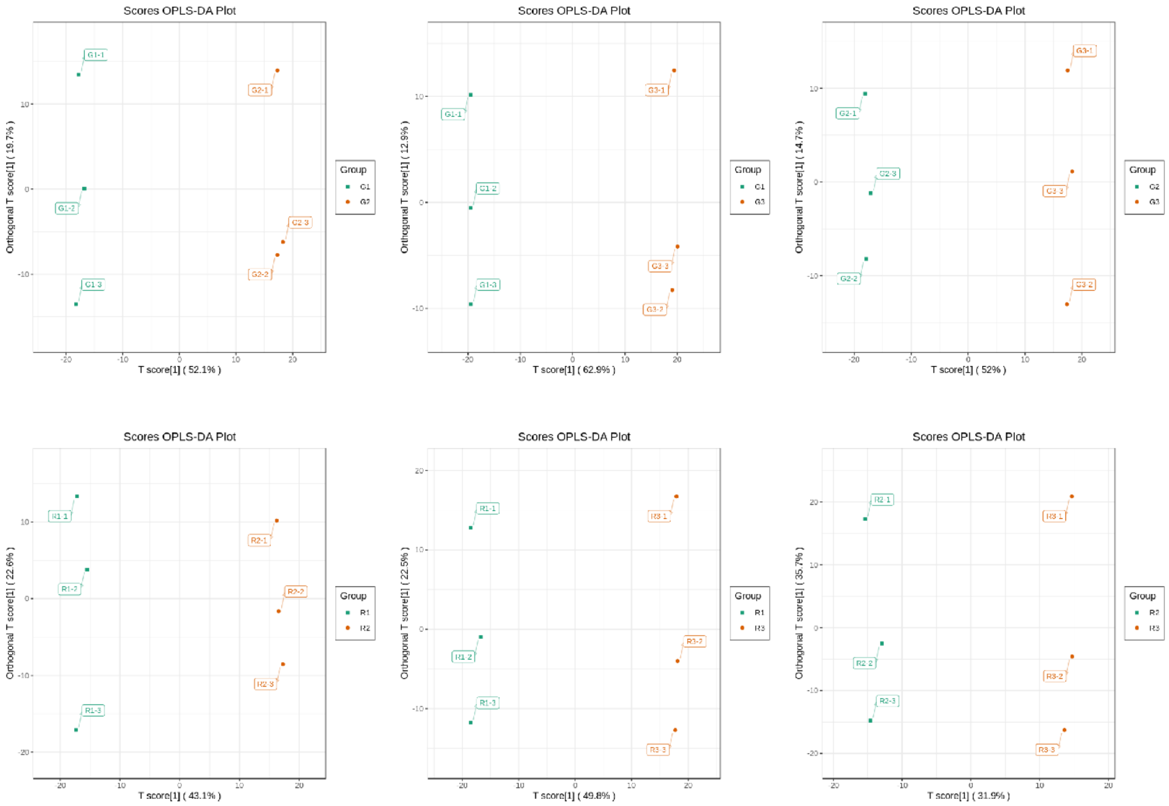

Figure S2. Orthogonal projections to latent structures discriminant analysis (OPLS-DA) for all samples.

Table S1. The content of terpenoids in different developmental stages of green and red prickly ash by GC-MS (mg/100g).

Compounds	R1	R2	R3	G1	G2	G3
D-Limonene	36.22	52.85	50.29	0	0	0
Bicyclo[3.1.0]hex-2-ene, 2-methyl-5-(1-methylethyl)-	0.02	0.32	0	0	0	0
ç-Terpinene	0.76	2.06	1.11	0	0	0
á-Myrcene	12.10	20.14	16.46	4.69	21.04	23.29
Bicyclo[3.1.0]hexane, 4-methylene-1-(1-methylethyl)-	1.21	4.81	3.31	0.17	2.42	3.58
Cyclohexene, 1-methyl-4-(1-methylethylidene)-	0.91	1.52	1.92	2.79	13.86	18.59
á-Ocimene	6.39	17.11	15.83	2.09	4.48	4.16
à-Phellandrene	1.00	3.43	3.98	0.59	1.18	1.54
à-Pinene	6.39	17.02	16.29	19.97	63.62	75.21
Terpineol	7.77	17.54	9.07	3.34	3.44	5.49
Citronellol	0.59	2.13	5.00	0	0	0
(S)-(-)-(4-Isopropenyl-1-cyclohexenyl)methanol	0.09	0	0	0	0	0
(-)-Myrtenol	0	0	0	1.23	1.06	0
p-Mentha-1,8-dien-7-ol	0	0.15	0.24	0	0	0
Terpinen-4-ol	10.89	12.96	7.97	0	0	0
2,6-Octadien-1-ol, 3,7-dimethyl-, (Z)-	0.41	0	0.00	0	0	0
Linalool	4.65	7.04	4.97	2.02	2.69	3.30
D-Verbenone	0.07	0.70	0.11	0	0	0
2-Cyclohexen-1-one, 3-methyl-6-(1-methylethyl)-	12.64	17.08	11.63	59.18	91.62	106.44
2-Cyclohexen-1-ol, 3-methyl-6-(1-methylethyl)-, acetate	0	0.87	0.61	0	0	0
à-Terpinyl acetate	25.52	36.24	24.59	0	0	0
2,6-Octadienal, 3,7-dimethyl-, (E)-	0	0	0.00	7.72	5.91	0
Limonene oxide, cis-	1.48	0.88	0.57	1.11	1.05	0.87
(-)-Germacrene-D	0.09	0.09	0.10	0	0	0
Azulene, 1,2,3,3a,4,5,6,7-octahydro-1,4-dimethyl-7-(1-methylethenyl)-, [1R-(1à,3aá,4à,7á)]-	4.19	1.48	0.65	0.30	0.44	0.33

(1R,2S,6S,7S,8S)-8-Isopropyl-1-methyl-3-methylenetricyclo[4.4.0.02,7]decane-rel-	1.32	3.14	2.83	6.01	33.22	27.57
à-Farnesene	0.69	1.83	1.30	0	0	0
1H-3a,7-Methanoazulene, 2,3,6,7,8,8a-hexahydro-1,4,9,9-tetramethyl-, (1à,3aà,7à,8aá)-	0.25	0	0.00	0	0	0
.alfa.-Copaene	0.43	0.70	0.78	0.46	0.69	0.55
Naphthalene, 1,2,3,4,4a,5,6,8a-octahydro-4a,8-dimethyl-2-(1-methylethenyl)-, [2R-(2à,4aà,8aá)]-	3.67	0	0.00	15.35	21.52	19.90
à-Muurolene	1.71	0.92	0.83	0.38	0	0
β-Elemen	20.05	23.36	15.54	37.15	55.96	54.16
Naphthalene, decahydro-4a-methyl-1-methylene-7-(1-methylethenyl)-, [4aR-(4aà,7à,8aá)]-	1.84	1.53	1.12	9.69	12.51	11.90
á-Guaiene	0.43	0.20	0.05	1.10	2.99	3.18
ç-Elemene	0.98	5.39	4.37	1.98	10.40	7.86
Naphthalene, 1,2,3,5,6,8a-hexahydro-4,7-dimethyl-1-(1-methylethyl)-, (1S-cis)-	3.28	3.87	3.04	1.93	2.88	2.12
Elemene isomer	0.64	3.50	2.22	3.39	7.30	6.08
Humulene	2.00	2.85	2.50	5.49	8.09	7.40
Caryophyllene	4.94	6.97	6.55	25.66	33.57	30.43
ç-HIMACHALENE	0.20	0.13	0.02	0.13	0.16	0.28
Ylangene	1.88	0.11	0.09	0.20	0.12	0.14
Muurolene	2.20	3.54	2.89	2.30	4.28	3.44
Longifolene-(V4)	0.02	0	0.00	1.15	0	0
Germacrene D	6.75	13.62	12.72	20.05	1.11	0
Aristolene epoxide	0.27	0	0.09	0	0	0
Copaene	0.35	0.39	0.12	0	0	0
à-Cadinol	0.06	0	0.00	0	0	0
(-)-Globulol	0.24	0	0.03	0.51	0.92	0.50

(3S,3aR,3bR,4S,7R,7aR)-4-Isopropyl-3,7-dimethyloctahydro-1H-cyclopenta[1,3]cyclopropa[1,2]benzen-3-ol	0.08	0.15	0.76	0.27	0.68	0.32
Cubenol	1.48	3.31	1.89	0.14	0.57	0.45
Globulol	0.46	1.96	2.42	0	0	0
Cyclohexanemethanol, 4-ethenyl-à,à,4-trimethyl-3-(1-methylethenyl)-, [1R-(1à,3à,4á)]-	1.88	1.10	0.00	0	0	0
2-Naphthalenemethanol, decahydro-à,à,4a-trimethyl-8-methylene-, [2R-(2à,4aà,8aá)]-	0.24	0.27	0.00	0	0	0
2-Naphthalenemethanol, 1,2,3,4,4a,5,6,7-octahydro-à,à,4a,8-tetramethyl-, (2R-cis)-	0.43	0.99	0.00	0.66	0	0
Nerolidol	0.21	0	0.00	0	0	0
2-((2R,4aR,8aS)-4a-Methyl-8-methylenedecahydronaphthalen-2-yl)prop-2-en-1-ol	0	1.02	1.56	0	0	0
Aromadendrene oxide-(2)	0	0.38	0	0	0	0
Caryophyllene oxide	0	0	0	0.69	0	0
Grindelic acid	0.90	0	0	0	0	0
cis-5,8,11,14,17-Eicosapentaenoic acid	0.06	0	0.06	0	0	0
trans-Geranylgeraniol	0	0	0	0	0	0.24
Arachidonic acid	0	0.27	0	0	0	0
1H-Naphtho[2,1-b]pyran, 3-ethenyldodecahydro-3,4a,7,7,10a-pentamethyl-, [3R-(3à,4aá,6aà,10aá,10bà)]-	0	0.02	0	0	0	0
Decanoic acid, decyl ester	0	0	0	0.56	0	0
Lupeol	0	0	0	0.08	0	0
Olean-12-ene-3,15,16,21,22,28-hexol, (3á,15à,16à,21á,22à)-	0	0	0	0	0.13	0
Lycoxanthin	0	0	0	0	0.30	0
Astaxanthin	0	0	0.03	0	0.08	0.06

Table S2. Terpenoids detected at different developmental stages of green and red Chinese prickly ash fruit by LC-MS.

Compounds	R1	R2	R3	G1	G2	G3
Cantharidin	19492.67±6518.07	18110.67±6041.05	17652.83±8769.23	73272.67±4635.32	73118.00±14045.94	84646.33±23567.80
Istanbulin A	21494.00±6490.38	9850.50±3975.52	2569.13±1099.73	49192.00±460.56	32991.00±3653.47	22262.00±1396.37
Aucubin	9.00±0.00	48784.00±17121.55	173946.67±35209.83	9.00±0.00	22011.67±3872.04	32050.00±4587.63
Jasminoside G	41351.33±20171.63	88859.00±32753.58	101677.67±20370.86	1055456.67±76810.35	1939500.00±382648.02	3193766.67±617150.69
Sweroside	287643.33±21967.26	496760.00±63580.29	521353.33±163832.72	432250.00±69550.07	647553.33±124170.83	730286.67±26442.36
Ixoroside	29921.00±2646.31	40163.33±5770.09	47795.00±4474.07	45743.67±10474.05	30229.67±3772.70	42672.00±4680.57
Gardoside	56458.00±25031.43	403513.33±112456.98	387433.33±136844.52	160783.33±23278.44	300793.33±22506.81	218766.67±17538.03
Geniposidic acid	60916.33±41565.02	396683.33±134380.24	389646.67±178866.54	161190.00±27166.01	354133.33±52637.01	252786.67±44704.71
Morroniside	20587.00±7864.52	63113.33±7577.98	119207.33±35968.61	207016.67±20314.66	262916.67±21639.66	351026.67±34315.86
Ixerisoside D	4824533.33±2609433.82	12829156.67±11070889.41	1901176.67±1866999.77	1425500.00±160645.67	664116.67±307379.34	339246.67±106132.31
Lupenone	9.00±0.00	324942.00±269341.35	736643.33±674849.61	9.00±0.00	9.00±0.00	9.00±0.00
24,30-Dihydroxy-12(13)-enolupinol	13128.07±4059.90	234166.67±109726.99	734353.33±278455.19	3295.77±1176.40	2911.83±1277.73	6227.93±1564.42
Betulinic acid	9.00±0.00	5771.20±2874.86	17237.30±7025.17	9.00±0.00	9.00±0.00	9.00±0.00
3,24-Dihydroxy-17,21-	9.00±0.00	12340.07±15218.38	9.00±0.00	21009.00±8099.45	21034.33±7077.15	20167.00±3523.19

semiacetal-12(13)oleanolic fruit						
2-Hydroxyoleanolic acid	1579.17±2719.61	10253.33±17743.71	9.00±0.00	21423.67±8291.84	18311.33±2529.13	22087.00±1479.30
Maslinic acid	2497.20±4309.69	13340.70±17576.35	9.00±0.00	15752.67±8067.84	11499.60±3593.72	13663.33±702.51

Noted: The value represents the relative value of the substance content.

Table S3. Genes of terpenoid synthesis pathway.

Gene Symbol	Description	ID
ACAT1	Acetyl-CoA C-acetyltransferase	EVM0095708
ACAT2		EVM0024272
HMGS1	Hydroxymethylglutaryl-CoA synthase	EVM0001928
HMGS2		EVM0067781
HMGS3		EVM0090351
HMGS4		EVM0094798
HMGS5		EVM0020657
HMGR1	3-hydroxy-3-methylglutaryl-coenzyme A reductase	EVM0026565
HMGR2		EVM0052782
MVK1	Mevalonate kinase	EVM0068292
MVK2		EVM0039066
PMK	Phosphomevalonate kinase	EVM0000069
MVD	Diphosphomevalonate decarboxylase	EVM0060324
IPI/IDI1	Isopentenyl-diphosphate Delta-isomerase	EVM0017527
IPI/IDI2		EVM0077335
IPI/IDI3		EVM0070462
IPI/IDI4		EVM0066063
GDS1	germacrene D synthase	EVM0091166
GDS2		EVM0012024
LUP	lupeol synthase	EVM0006329
DXS1	1-deoxy-D-xylulose-5-phosphate synthase	EVM0000221
DXS2		EVM0033747
DXS3		EVM0013204
DXS4		EVM0062949
DXS5		EVM0014177
DXR1	1-Deoxy-D-xylulose 5-phosphate reductoisomerase	EVM0071397
DXR2		EVM0039865
CMK	4-diphosphocytidyl-2-C-methyl-D-erythritol kinase	EVM0016577
HDS1	4-hydroxy-3-methylbut-2-en-1-yl diphosphate synthase	EVM0037791
HDS2		EVM0079161
HDS3		EVM0094256
HDS4		EVM0042921
HDR1	4-hydroxy-3-methylbut-2-enyl diphosphate reductase	EVM0049200
HDR2		EVM0057408
FDPS1	Farnesyl diphosphate synthase	EVM0022266
FDPS2		EVM0091963
FDPS3		EVM0095854
LIS1	Linalool synthase	EVM0068565
LIS2		EVM0070978
TOT1	Tocopherol O-methyltransferase	EVM0011066

TOT2	EVM0046896
